# Improving the usability of online resources to support implementation research and practice: The RE-AIM website use case

**DOI:** 10.1017/cts.2025.10070

**Published:** 2025-06-18

**Authors:** Emiliane L. Pereira, Thomas E. Strayer, Samantha M. Harden, Russell E. Glasgow, Christina Studts, Paul A. Estabrooks, Amy G. Huebschmann

**Affiliations:** 1 University of Nebraska Medical Center College of Public Health, Department of Health Promotion, Omaha, USA; 2 Vanderbilt University Medical Center, Pulmonary Circulation Center, Division of Allergy, Pulmonary and Critical Care Medicine, Nashville, Tennessee, USA; 3 Virginia Tech, Department of Human Nutrition, Foods and Exercise, Blacksburg, USA; 4 University of Colorado Anschutz Medical Campus (CU-SOM), Department of Family Medicine, Aurora, USA; 5 CU-SOM, Adult and Child Center for Outcomes Research and Delivery Science (ACCORDS), Aurora, USA; 6 CU-SOM, Department of Pediatrics, Aurora, USA; 7 University of Utah, Department of Health & Kinesiology, Salt Lake city, Utah, USA; 8 CU-SOM, Department of Medicine, Division of General Internal Medicine, Aurora, USA; 9 CU-SOM, Ludeman Family Center for Women’s Health Research, Aurora, USA

**Keywords:** Dissemination and implementation science, education, usability, online resources, translational research, mixed-methods, RE-AIM

## Abstract

**Introduction:**

Our overall goal was to enhance the usability and interactivity of the RE-AIM website (re-aim.org) and improve resources to support the application of the RE-AIM framework within the context of dissemination & implementation (D&I) research and practice.

**Methods:**

We applied a mixed-methods approach to obtain user feedback from 24 D&I researchers and practitioners. Usability (System Usability Scale) and interactivity (Interactivity Scale) were assessed through validated surveys, at baseline and after two iterative rounds of website modifications (Phase 1 and Phase 2). We also conducted qualitative assessments at each phase.

**Results:**

Qualitative baseline and Phase 1 findings indicated a need to simplify organization, enhance information accessibility, provide concrete guidance on applying RE-AIM, and clarify contextual factors related to RE-AIM constructs. After streamlining website and homepage organization, Phase 2 qualitative results suggested improved user navigation experience; users also requested greater interactivity. Modifications included: new interactive planning tool and a video introduction of contextual factors influencing RE-AIM outcomes. Significant improvements were found in the SUS score from baseline to Phase 1(64.2[SD18.7] to 80.8 [SD 12.1] (*p* < .05) and remained higher in Phase 2(77.1[SD 15] (*p* = 0.08). Interactivity also improved from baseline to Phase 2(3.5[SD1.2] to 41[0.9], though not statistically significant.

**Conclusion:**

User-centered feedback on online resources, as exemplified by this use case example of enhancements to the RE-AIM website, are important in bridging the gap between research and practice, and the revised website should be more accessible and useful to users.

## Introduction

There is a rapidly growing demand for training and resources to enhance capacity in understanding and utilizing Dissemination and Implementation (D&I) frameworks and methods. As D&I frameworks form the foundation for D&I research and practice, it is crucial to provide guidance on their application [1]. There is a need to develop resources for two distinct audiences: D&I researchers and D&I practitioners [2]. These two audiences are differentiated by the fact that D&I researchers seek to advance the methods and theoretical understanding of D&I, whereas implementation practitioners are concerned with pragmatic implementation of evidence-based interventions in clinical practice or public health settings [2,3]. The distinction is relevant as D&I researchers and practitioners may have different preferences and priorities for accessing D&I resources [4–6].

Thus, providing D&I resources relevant to both researchers and practitioners is important as is the need to address their perspectives and preferences. This is in line with the guidance from Brownson et al. (2018) that highlighted the channel of communication used is a key factor to consider when distributing D&I resources and tools to specific audiences [7]. There is a demand for technology-facilitated channels beyond formal D&I trainings and peer-reviewed publications to foster the utilization of D&I tools in implementation research and practice [1]. Existing technology-facilitated resources that enhance the understanding of D&I frameworks include, among others: the Dissemination and Implementation Models in Health Research website [8], the Society for Implementation Research [9], the Center for Implementation [10], the University of Colorado ACCORDS DIS Program website [11], the Normalization Process Theory website [12], the Consolidated Framework for Implementation website [13], the National Cancer Institute [14], and the EPIS Implementation Framework [15].

One of the longest-standing web-based resources for D&I is the Reach, Effectiveness, Adoption, Implementation, and Maintenance (RE-AIM) website [16]. This website, developed by the National RE-AIM/PRISM Consortium, is dedicated to implementing a robust and evolving framework (RE-AIM) that advances science, enhances practice, and influences policy through collaboration and training [17]. To achieve this purpose, the website provides guidance on use of the RE-AIM framework for researchers, students, practitioners, and community leaders. When this project began, the initial version of the RE-AIM website included a welcome landing page, a list of professional education webinars, a publication library, definitions of framework dimensions, and resources and tools (e.g., calculators, checklist, measures, figures and tables) [17].

Although the RE-AIM website has undergone several iterations over the past 20 years, primarily focusing on keeping resources up to date for D&I audiences, the website design, organization, and navigation largely remained largely unchanged. Given the limited funding and organizational capacity, these past website revisions were conducted with very limited input from website end users (i.e., reactive responses to user emails identifying broken weblinks). Incorporating user perspectives and feedback into the RE-AIM website could address the current calls within the D&I field to enhance resources and tools for a diverse audience, including students, researchers, clinicians, and practitioners [18]. The purpose of this project was to: (1) conduct a systematic mixed methods evaluation of the RE-AIM website (re-aim.org) usability needs and priorities among two types of end-users: D&I researchers and implementation practitioners; (2) iteratively adapt the RE-AIM website based on user testing, allowing for input and guidance from end-users and RE-AIM experts, and (3) compare RE-AIM website usability before and after adaptations.

## Methods

We used a convergent mixed-methods approach [19] to assess the usability of the available resources on the RE-AIM website at baseline and after two phases of website adaptations. This approach involved the simultaneous collection of both qualitative and quantitative data. Specifically, we utilized quantitative usability scores to guide our qualitative investigation and understand users’ experiences and perceptions. The website adaptation process was overseen by seven members of the National RE-AIM/PRISM Consortium [20] with expertise in D&I and one website design consultant. The adaptation process involved evaluating usability and interactivity at baseline, followed by Phase 1 assessment after initial updates, and finally Phase 2 assessment after final updates. Data collection took place between March, 2020, and September, 2021, following approval by the University of Colorado Institutional Review Board.

### Participants

Eligible participants for user testing in this study were behavioral or D&I researchers, D&I practitioners (including clinicians), and graduate students. We employed convenience and snowball sampling to recruit via email participants from a contact network list (*n* = 22) provided by National RE-AIM/PRISM Consortium as well as participants’ social networks. The list included a diverse group of researchers and clinicians, all trained in D&I and highly familiar with RE-AIM.

### Quantitative assessment

The quantitative assessment of the study utilized a 25-item survey questionnaire that combined the System Usability Scale (SUS) [21] and the Interactivity Scale [22]. The SUS scale is a widely validated measure used for over 30 years, and it has demonstrated high reliability, with Cronbach’s alpha ranging from 0.85 to 0.91 and strong positive correlation with other established measures (*r* = 0.81) (Bangor et al., 2008). It includes 10 standardized items assessing effectiveness, efficiency, and user satisfaction, with SUS scores ranging from 0 to 100. A score of 68 represents average usability and below 60 suggests poor usability [23]. The Interactivity Scale [22], which measures participation, information flow, and interaction speed, also has demonstrated high reliability, with Cronbach’s alpha ranging from 0.79 to 0.96 [24] . This scale uses a 5-point Likert scale, ranging from “strongly disagree” (1) to “strongly agree” (5). Higher scores on the Interactivity Scale reflect greater user engagement, ease of navigation, effective information flow, and fast response times.

### Qualitative assessment

Semi-structured interviews were conducted to gather users’ feedback using a “think aloud” [25] process across assessments. Each interview session, conducted via Zoom, lasted approximately 30 to 40 minutes. Participants shared their screen while navigating the RE-AIM website to complete predefined tasks. Participants were encouraged to express their thoughts aloud regarding the website’s usability, organization, clarity, and any navigation challenges they encountered during the task completion. All participant interviews were video recorded to facilitate further analysis. The interview guide was developed in collaboration with the RE-AIM working group. It consisted of three sequential sections: 1) navigation through the RE-AIM website using the “think-aloud” process, where participants were prompted with specific questions; 2) semi-structured interview questions to gather additional insights from participants; and 3) a Qualtrics survey link to complete the survey measures. Throughout the assessment phases, the National RE-AIM/PRISM Consortium carefully considered user feedback critiques and implemented key improvements in response to them.

### Data analysis

The survey results were exported from Qualtrics and analyzed using IBM SPSS Statistics for Windows, version 28. SUS scores and interactivity scores were calculated for each participant, and descriptive statistics (means and standard deviations) were computed for the sampled populations in each study assessment (baseline, Phase 1, and Phase 2).

Transcriptions of the semi-structured interviews were generated using the Otter.ai website. Thematic codes were developed through an inductive process by the lead author (EP) and refined through discussions with the expert website team. These codes were based on participants’ key points regarding usability, as expressed during the interviews, in a qualitative content analysis approach. NVivo software was used to assist with categorization of themes.

## Results

A total of 24 participants completed the interviews and surveys for this study. At baseline, there were 9 participants, followed by 9 participants in Phase 1 and 6 participants in Phase 2. There was a mix of new participants and those who continued to participate across the rounds. The participants’ roles and backgrounds varied across the different phases. In baseline, the participants (*n* = 9) consisted of D&I researchers who possessed a high level of familiarity with the RE-AIM framework. Phase 1 included researchers (*n* = 9) at different career stages, such as graduate students and faculty, with varying levels of expertise in D&I. Lastly, Phase 2 (*n* = 6) comprised (D&I) researchers and clinicians who regularly implement programs within health systems.

### System usability

**Figure 1.**
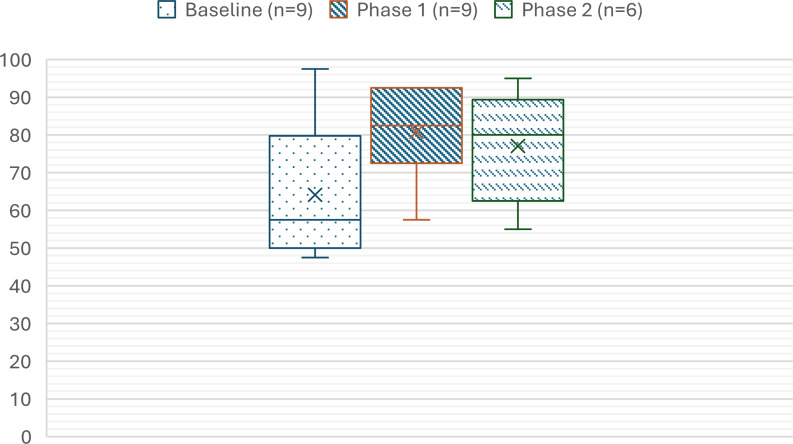
Summary System Usability Scores across rounds.

The SUS mean scores for assessment are summarized in Figure 1. The results indicate significant improvement in usability from baseline (64.2[SD 18.7]) to Phase 1 (80.8 [SD12.1], *p* < 0.05) and it remained within a good range between Phase 1 and Phase 2 (77.1[SD 15], *p* > 0.05). Noteworthy sub-items of the SUS that exhibited substantial improvement (∼1 point) from baseline to Phase 2 include lower scores for the reverse-scored item 2 (“I found the system unnecessarily complex”), higher scores for item 5 (“I found the various functions in this system were well integrated”), and lower scores for item 8 (“I found the system very awkward to use”). Across all SUS items, statement 1 (“I think that I would like to use this system frequently”) received the most favorable usability responses, with a mean score of 4.35 (see Table 1).

**Table 1.** System Usability Score (SUS) statements across assessments

	SUS scale items	Baseline (*n* = 9)	Phase 1 (*n* = 9)	Phase 2 (*n* = 6)	Baseline vs Phase 1	Baseline vs Phase 2
		Mean (SD)	Mean (SD)	Mean (SD)	*p*-value	*p*-value
	Total mean score	3.4 (1.2)	4.0 (1.1)	3.9 (1.2)	<0.001	0.013
1	I think that I would like to use this system frequently.	4.0 (0.8)	4.5 (0.5)	4.5 (0.5)	0.06	0.117
2	I found the system unnecessarily complex.*	3.2 (1.2)*	2.3 (1.3)*	2.1 (1.1)*	0.07	0.058
3	I thought the system was easy to use.	3.3 (1.0)	4.4 (0.7)	3.5 (1.2)	0.008	0.388
4	I think that I would need the support of a technical person to be able to use this system.*	1.2 (0.4)*	1.5 (0.5)*	1.0 (0.0)*	0.082	0.058
5	I found the various functions in this system were well integrated.	3.1 (1.2)	4.1 (0.6)	3.6 (1.3)	0.024	0.122
6	I thought there was too much inconsistency in this system.*	2.7 (1.2)*	2.3 (0.8)*	2.5 (1.3)*	0.191	0.343
7	I would imagine that most people would learn to use this system very quickly.	3.6 (1.0)	4.1 (0.6)	4.1 (0.7)	0.135	0.159
8	I found the system very awkward to use.*	3.0 (1.2)*	1.3 (0.5)*	1.8 (1.1)*	<0.001	0.044
9	I felt very confident using the system.	3.8 (0.7)	4.4 (0.7)	4.3 (0.5)	0.069	0.122
10	I needed to learn a lot of things before I could get going with this system.*	2.1 (1.0)	1.7 (1.0)*	1.8 (1.1)*	0.26	0.32

These 5-point Likert scale ratings included both *reverse scored (negative tone) items (lower is better) and positively tone items (higher is better). Total mean score calculated by averaging the mean scores of positive and reverse-scored negative tone items.

Table 2 presents responses to interactivity mean scores. Although there were no statistically significant differences in interactivity scores across phases, mean scores showed improvement in each of the three dimensions of interactivity: 1) active control; 2) two-way communication; and 3) synchronicity. The active control dimension of usability received the highest scores, with an overall mean of 4.4, followed by synchronicity with a mean of 4.0. In contrast, items focused on two-way communication received the lowest scores, with a mean of 3.0, suggesting the need for future work to improve this area.

**Table 2.** Interactivity scale

	Scale items	Baseline (*n* = 9)	Phase 1 (*n* = 9)	Phase 2 (*n* = 6)
		Mean (SD)	Mean (SD)	Mean (SD)
	Total mean score	3.5 (1.2)	3.8 (1.0)	4.1 (0.9)
	*Active control*			
1	I felt that I had a lot of control over my visiting experiences at this website.	4.0 (1.0)	4.4 (0.7)	4.6 (0.8)
2	While I was on the website, I could choose freely what I wanted to see.	4.5 (0.5)	4.6 (0.7)	4.8 (0.4)
3	While surfing the website, I had absolutely no control over what I can do on the site.*	1.5 (0.5)	1.4 (0.5)	1.1 (0.4)
4	While surfing the website, my actions decided the kind of experiences I got.	4.0 (0.7)	4.2 (0.6)	4.1 (0.7)
	*Two-way communication*			
5	The website is effective in gathering visitors’ feedback.	2.7 (1.2)	2.5 (1.1)	3.0 (0.0)
6	This website facilitates two-way communication between the visitors and the site.	2.5 (1.0)	3.3 (1.2)	3.1 (0.7)
7	It is difficult to offer feedback to the website.*	3.1 (1.0)	3.1 (1.1)	2.6 (1.0)
8	The website makes me feel it wants to listen to its visitors.	2.5 (1.1)	3.0 (0.8)	3.5 (0.5)
9	The website does not at all encourage visitors to talk back.*	3.1 (0.9)	3.2 (0.8)	2.5 (0.5)
10	The website gives visitors the opportunity to talk back.	2.7 (0.8)	3.1 (0.7)	3.0 (0.6)
	*Synchronicity*			
11	The website processed my input very quickly.	4.0 (1.1)	3.8 (0.6)	4.3 (0.8)
12	Getting information from the website is very fast.	4.0 (1.1)	4.4 (0.5)	4.6 (0.5)
13	I was able to obtain the information I want without any delay.	3.7 (1.3)	4.3 (0.7)	4.6 (0.5)
14	When I clicked on the links, I felt I was getting instantaneous information.	3.6 (1.5)	4.4 (1.0)	4.6 (0.5)
15	The website was very slow in responding to my requests.*	2.0 (1.1)	1.7 (0.9)	1.0 (0.0)

These 5-point Likert scale ratings included both *reverse scored (Negative tone) items - lower is better, and Positive tone items. Total mean score calculated by averaging the mean scores of positive and reverse-scored negative tone items.

### Qualitative assessment

Key themes were generated from the analysis of the “think aloud” interview transcripts for each Phase, and certain themes were revisited across phases. At a high level, themes from the baseline participants emphasized issues such as confusion, missing information, and duplication. In Phase 1, end users positively commented on the simplicity of the website organization and expressed a desire for more clarity regarding PRISM and RE-AIM integration. Phase 2 feedback highlighted the positive reception of the homepage welcome video, the new tour guide, and the newly implemented features.

### Baseline feedback

At baseline, four main themes emerged: 1. Acceptability (exemplified by terms such as “like” or “do not like”); 2. Navigability (exemplified by comments of “confusing” or “missing”); 3. End users’ priority (exemplified by comments of “features would like to see available”) and 4. Recommendations. In terms of Acceptability, website factors that participants “liked” included the amount of information, tools, and publications available on the website. There was an impression that the information was highly relevant to those in the D&I field. However, participants sometimes felt overwhelmed by the amount of text on certain sub-pages of the site. In terms of Navigability, end users at baseline found that information was often duplicated in multiple places, leading to confusion in website navigation. End users also found the integration of components across different sections to be inadequate, making difficult to understand how the sections connected with each other. Additionally, end users felt confused by the overall structure of the website and weren’t sure where to go for the resources they wanted.

### Detailed description of website revisions after baseline feedback

After reviewing the feedback provided during baseline, the website National RE-AIM/PRISM Consortium generated multiple potential solutions, and initial areas to revise were rated in terms of 2 factors: feasibility of making changes in terms of both consortium effort and web designer costs, and projected impact of making these improvements (see Table 3). Upon review of these ratings of effort, cost, and impact, the working group decided on the following changes: 1) restructure the website in a more organized and visually appealing manner, including a redesigned homepage and site map, 2) creating a sub-page with improved access to publications and presentations, 3) improved interactivity of the resources (e.g., editable checklists, interactive planning tools), 4) concrete guidance of the use of the RE-AIM framework for planning, implementation, and evaluation purposes.

**Table 3.** Baseline recommendations

Recommendations	Efforts	Cost	Impact	Website adaptations
*Repackage the website to deliver information in a more organized and visual manner (e.g., make the homepage look less overwhelming, categorize different sections for end users (e.g., publications, presentations)	Medium	Low	High	Implemented in Phase 1: Homepage restructured with creation of three new sections: “Learn,” “Apply” and “High priority resources”
*Create a database system to easily list out and access all RE-AIM publications and presentations	Low	Low	High	Implemented in Phase 1: New searchable database for publications.
Increase the interactivity of the resources such as tools and checklists available (e.g., provide more editable documents to download and fill out)	High	Low	High	Implemented in Phase 2: Interactive tool, editable pdfs.
*Provide more concrete guidance on use of the RE-AIM framework for planning or evaluation purposes	High	Low	High	Implemented in Phase 2: Expanded guidance adding common misconceptions related to RE-AIM.
Give examples of grants using RE-AIM framework	Medium	Low	Medium	Implemented in Phase 2: Grant-writing resources section added.
*Facilitate two-way communication between end users and the website: more ways for end users to provide feedback	Low	Low	Low	Not fully implemented. Considered for future updates.
Create a search box separated by categories to make it easier for end users to find information available on the website with fewer clicks	Medium	Medium	Low	Not fully implemented. Considered for future updates.
*Check that all the links are working perfectly and leading to the information expected	Medium	Low	Low	Implemented in Phase 1: Links verification and correction.

*Designates recommendations addressed in Phase 1: website reorganization, database system development, addition of checklists, provision of more detailed guidance, inclusion of grant examples, and verification of links. Abbreviations: RE-AIM= Reach, Effectiveness, Adoption, Implementation, and Maintenance.

### Phase 1

The themes from the initial baseline qualitative analysis were identified again in qualitative analysis of the think aloud process from Phase 1, including: acceptability, navigability, end users’ priority, and recommendations. In terms of acceptability, end users reacted positively to the visual simplicity and colorfulness of the revised website homepage. In terms of navigability, end users had mixed responses with some positives and some negatives – while they found it easy to go to the “Apply” section when wanting to use RE-AIM for a current project, they expected to find “Resources” in a stand-alone section rather than within a sub-page of Apply. In terms of priority, end users appreciated access to example grants using RE-AIM but wanted more details on those grants. While they appreciated having more accessible information about how the RE-AIM outcomes and PRISM contextual factors fit together, they were not fully clear on the relationship between PRISM and RE-AIM, and why PRISM was being described on the landing page of this website. For the “Learn” section, the end users liked the structure and how it included content for RE-AIM and PRISM individually. The new fillable RE-AIM checklist received positive feedback, and participants recommended similar interactive features like that available throughout the whole website (see Table 4).

**Table 4.** Overview of Phase 1 results

Themes	Participants quotes
*Homepage organization	“Liked the simplicity and colorful page” (Acceptability) “Liked the organization and the colors” (Acceptability) “The page needs to have more information why PRISM is here” (Acceptability)
*Grant writing resources	“It was not intuitive where to find Grant Writing resources” (Navigability) “Too many layers to find grant writing resources here” (Navigability) “Very nice example of grants” (Priority, Acceptability) “Really helpful for people” (Priority)
Learn section organization	“Very clean” (Acceptability) “Liked the learn section because it’s structured (and) has RE-AIM and PRISM” (Acceptability, Priority) “Would be nice to have something (like the) RE-AIM checklist for all the other pieces as well” (Priority for interactivity)
*Resources and Tool section	“Some resources are repeating, I do not know why this is here” (Navigability) “It is not aligned in the same order” (Navigability)
Key issues	“Rethinking where the things actually sit is the biggest issue” “Whatever the reason to have PRISM (is), that needs to be carried throughout the whole website”

*Updated for Phase 2 based on this feedback: new homepage featuring a website tour video, updated development grant resources section, and dedicated session for PRISM and guidance utilization. Abbreviations: PRISM, Practical = Robust Implementation and Sustainability Model;RE-AIM = Reach, Effectiveness, Adoption, Implementation and Maintenance.

### Phase 2

In Phase 2, the comments were summarized following the same pattern as phase 1, according to the tasks conducted with the participants and included the new features and improvements implemented during this phase (see Table 5 for themes). The new improvements after Phase 1 included: “Start here” button on the Home Page (link to a website site map and tour guide video), a welcome video on the Home Page, a video on the home page describing how RE-AIM and PRISM fit together, and a new interactive planning tool. Feedback included: the website tour guide was appreciated by the end users as an option, although most participants did not spend time watching it. Results related to the “welcome video” and “RE-AIM and PRISM fit together” video found that the tools were helpful for end users; their preference was for shorter videos of less than one minute, although they found more interesting and relevant content in the longer video (“How RE-AIM and PRISM fit together). The new interactive tool was considered helpful and a great tool to organize people’s thoughts as one more resource to improve website interactivity.

**Table 5.** Phase 2 results

Themes	Participants quotes
Homepage organization	“I love this logo” (Acceptability) “The color and the context look pretty good” (Acceptability) “It looks super professional” (Acceptability) “It looks a little nonstandard from some websites that I typically navigate” (Acceptability - negative)
Website tour	“I usually do not do that, although sometimes I should” (Acceptability, Navigability) “It is nice (to) take a tour” (Navigability)
Grant writing resources	“I think having something like this is really, really important” (Priority) “This is helpful. I just do not like the layout” (Priority, Acceptability) “A table you’ll be more useful” (Acceptability - negative)
Learn section organization	“Examples are good” (Acceptability) “It is very long in detail” (Acceptability – negative) “Frequently asked questions are excellent” (Acceptability) “Might be nice to have a spot for people enter their questions, so that maybe this becomes more iterative overtime” (Acceptability, Priority)
Resources and Tool section organization	“I think that’s super helpful” (Acceptability, Priority) “I think it’s great that you have all those tools” (Acceptability, Priority) “I think the organization looks fine. I do not know when I would go back here to look at this stuff. Unless, unless, you know, there was something I was specifically looking for” (Acceptability, Priority)
Interactive planning tool usability	“This interactive tool is helpful” (Priority) “I think that this is interesting, but like it’s pretty limited space” (Acceptability – negative) “A document with everything kind of summarized for you. Which I think is really cool. It helps organize thoughts” (Priority, Acceptability)
Video acceptability	“How RE-AIM and PRISM fits together video. This might be interesting” (Acceptability) “I love that this one’s only a minute. Small videos, not long ones” (Acceptability) “Welcome video- I do not know if I would watch that. I mean, I appreciate I think, I guess I’m a little more of a text user” (Acceptability) “I think the video is helpful because he kind of talks about what the webpage is supposed to do” (Acceptability)

Abbreviations: PRISM=Practical, Robust Implementation and Sustainability Model;RE-AIM= Reach, Effectiveness, Adoption, Implementation, and Maintenance.

## Discussion

This convergent, 3-phase mixed-methods evaluation of end users’ feedback significantly enhanced the usability of the RE-AIM website, a prominent online resource to support the application of D&I science methods. Among our target audience of D&I researchers and implementation practitioners, the average SUS significantly improved; the mean SUS score in the final Phase and across all phases (80.8 and 74.0, respectively) surpassed the literature’s SUS threshold score of usability of 68 [14]. The website’s interactivity rating also showed a signal towards improvement (albeit not statistically significant). These findings highlight the successful potential of this human-centered design approach to enhance ours and other D&I resource websites’ overall usability.

Qualitative analyses identified key themes of acceptability, navigability, end users’ priority, and recommendations for better organization of the website through the use of new website sections and sub-headings. The combination of video and text summaries of resources in each website section were specific organizational recommendations that seemed to drive higher usability scores. These themes provided valuable insights into the elements that were crucial for improving the usability of the RE-AIM website. The qualitative themes suggested that end users prioritize more interactive resources that elicit their input and provide structured feedback. Even automated interactivity, such as the interactive planning guide, was well-received by end users. These findings serve as a case example that can inform the enhancement of other D&I online resources, offering valuable guidance to the field.

Investigating the sub-items of the SUS alongside the qualitative data, the study found that those related to navigability, such as confusing navigation, integration of components, and awkwardness of navigation, showed the most improvement across all phases. End users valued the website’s prioritization of critical content, including access to essential resources for D&I research and practice. The reorganization of the site structure and highlighting of key resources played a crucial role in driving the significant improvements in these specific SUS sub-items, ultimately contributing to the overall enhancement of SUS scores.

There was some variability across subscales for the Interactivity ratings. The relatively high ratings on the Interactivity Scale, particularly on the “Active Control” subscale showed that the RE-AIM website provided an experience where end users could choose information and receive quick responses (mean: 4.75). The website’s degree of active control contributed to its usability and interactivity. However, two-way communication, a manifestation of social interactivity, received lower scores compared to other Interactivity dimensions. Lack of improvement of interactivity over the phases may have been partly related to ceiling effect for certain domains and the limited two-way communication.

Through semi-structured interviews and the “think aloud” process, participants expressed appreciation for the abundance of information and resources on the RE-AIM website. However, negative feedback highlighted duplicative information, section categorization issues, and the lack of guidance on integrating RE-AIM with PRISM. This study’s website improvement process addressed these specific concerns, resulting in a more cohesive website that better supports end users in effectively navigating the RE-AIM framework and its integration with PRISM. The iterative approach to incorporating end users’ feedback was instrumental in identifying and remedying these issues, ultimately enhancing the website’s overall usability.

### Strengths and limitations

A strength of this study is taking a user-centered design and mixed-methods approach to guide revisions to an online tool for researchers: the RE-AIM website. Limitations include a relatively small sample size (*n* = 24) and convenience sampling of researchers and practitioners with familiarity of the RE-AIM framework, which may not fully represent all end users of the website.

### Implications/future research

Online resources are paramount for the dissemination and implementation of relevant frameworks and interventions. The study offers valuable insights into improving the usability and interactivity of the RE-AIM website as an example of D&I online resources. It highlights several key factors from experts and end-users with diverse field experiences. These factors should be further explored by other D&I online resources to determine commonalities and unique challenges. The identified factors affecting usability and end users’ preferences for interactivity should help inform the development of future D&I online resources. Features like interactive tools, sub-menus for high-priority resources, and highlighting key aspects of the D&I framework are considered valuable. Future research could evaluate other D&I website online resources using similar user-centered design and assessment methods to further enhance other D&I resources.

## Conclusion

User-centered feedback on online resources, as exemplified by this use case example of enhancements to the RE-AIM website, are important in bridging the gap between research and practice. The improvements to the RE-AIM website should enhance the usefulness and usability of the website and have implications for the broader D&I field and should enable a broader and more diverse audience to embrace and effectively utilize the RE-AIM framework and its PRISM expansion[26]. Incorporating the perspectives of end users made the website better align with the needs and priorities of researchers, practitioners, students, and public health professionals, making it a more powerful tool for translating evidence into action.

## Data Availability

The datasets used and/or analyzed during the current study are available from the corresponding author on reasonable request.
